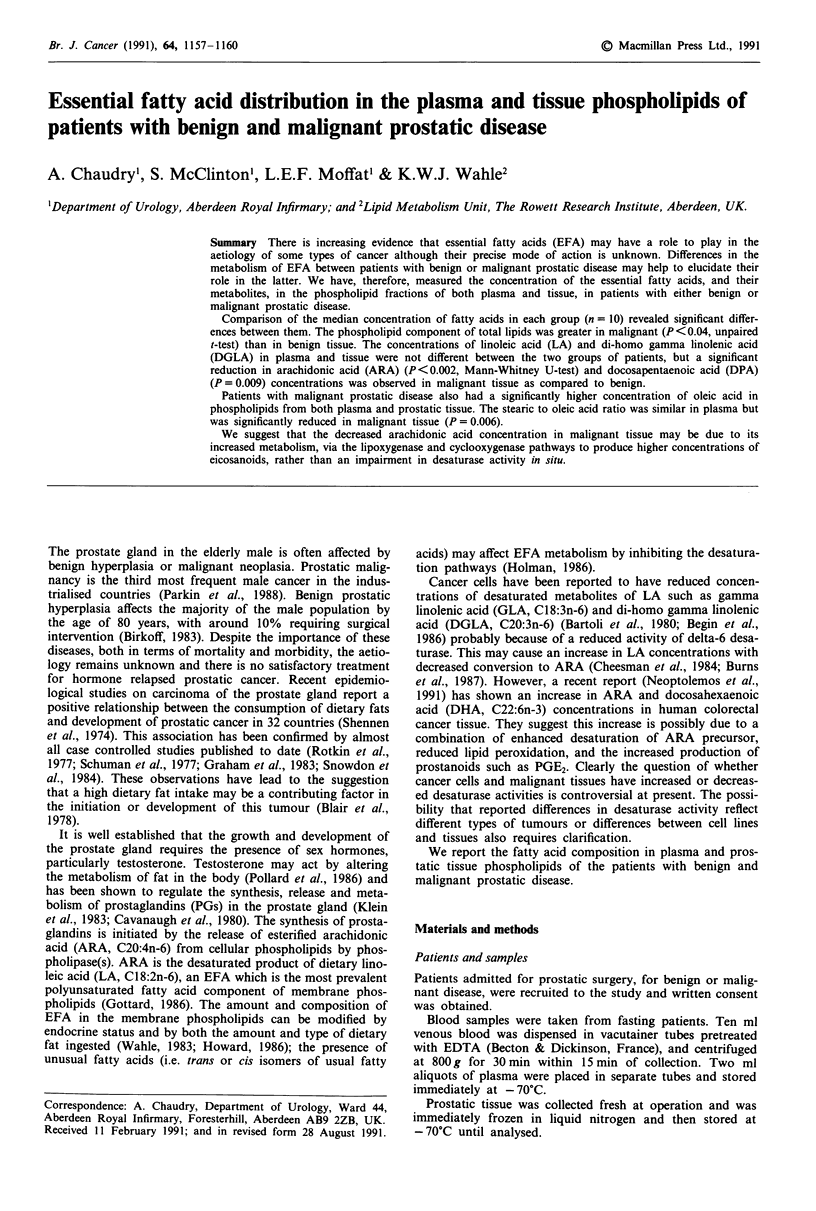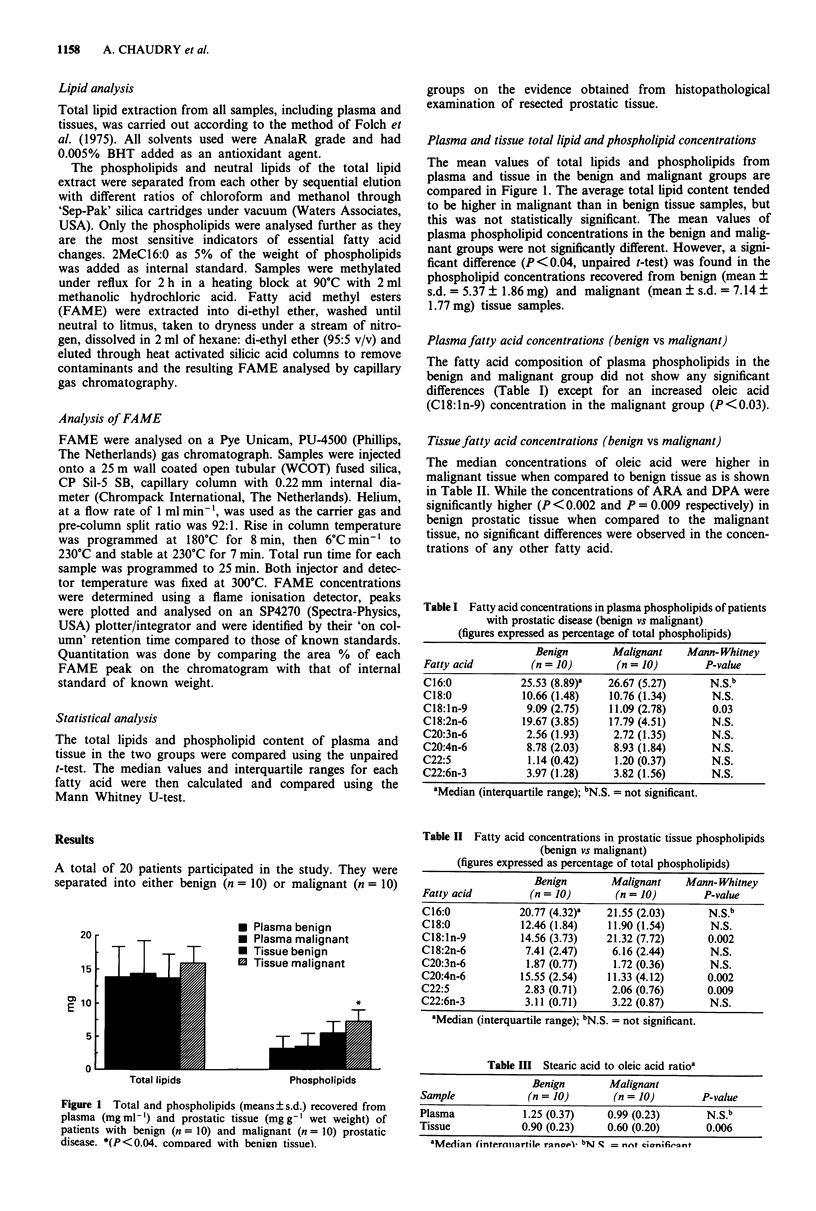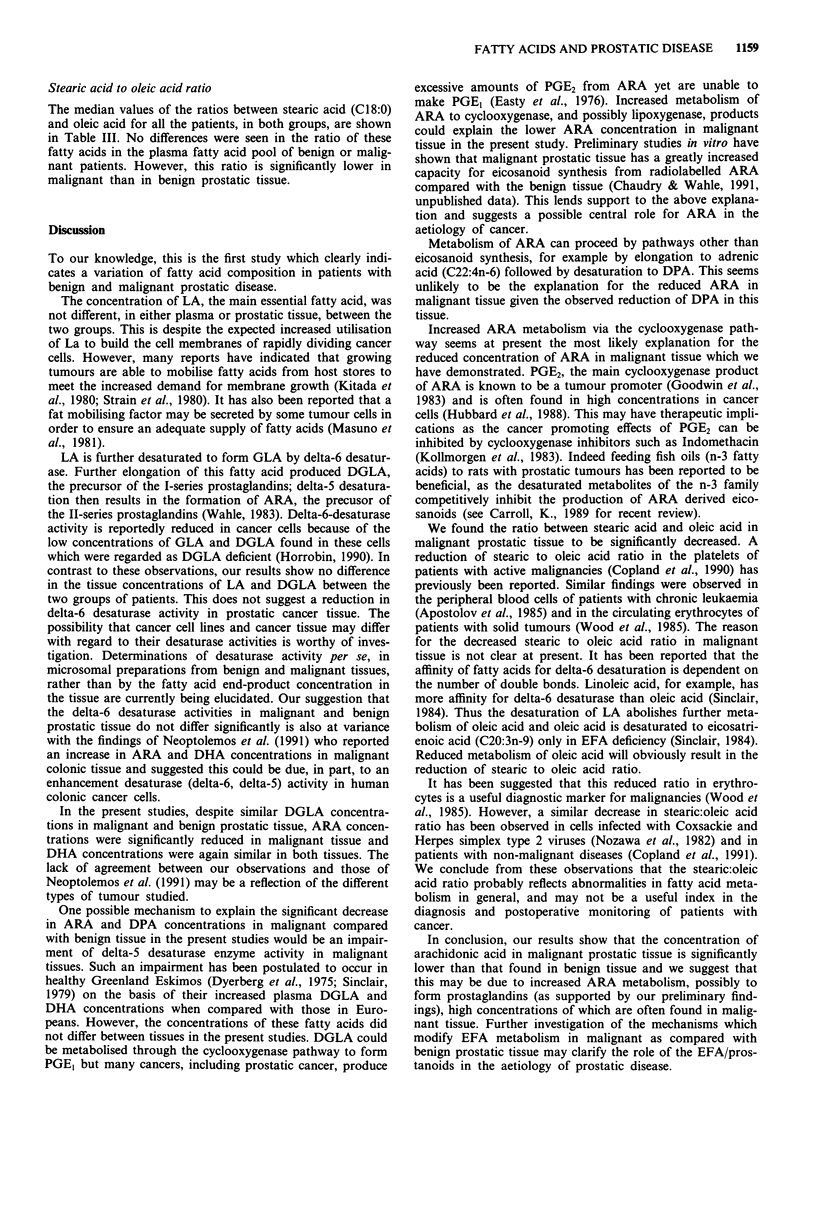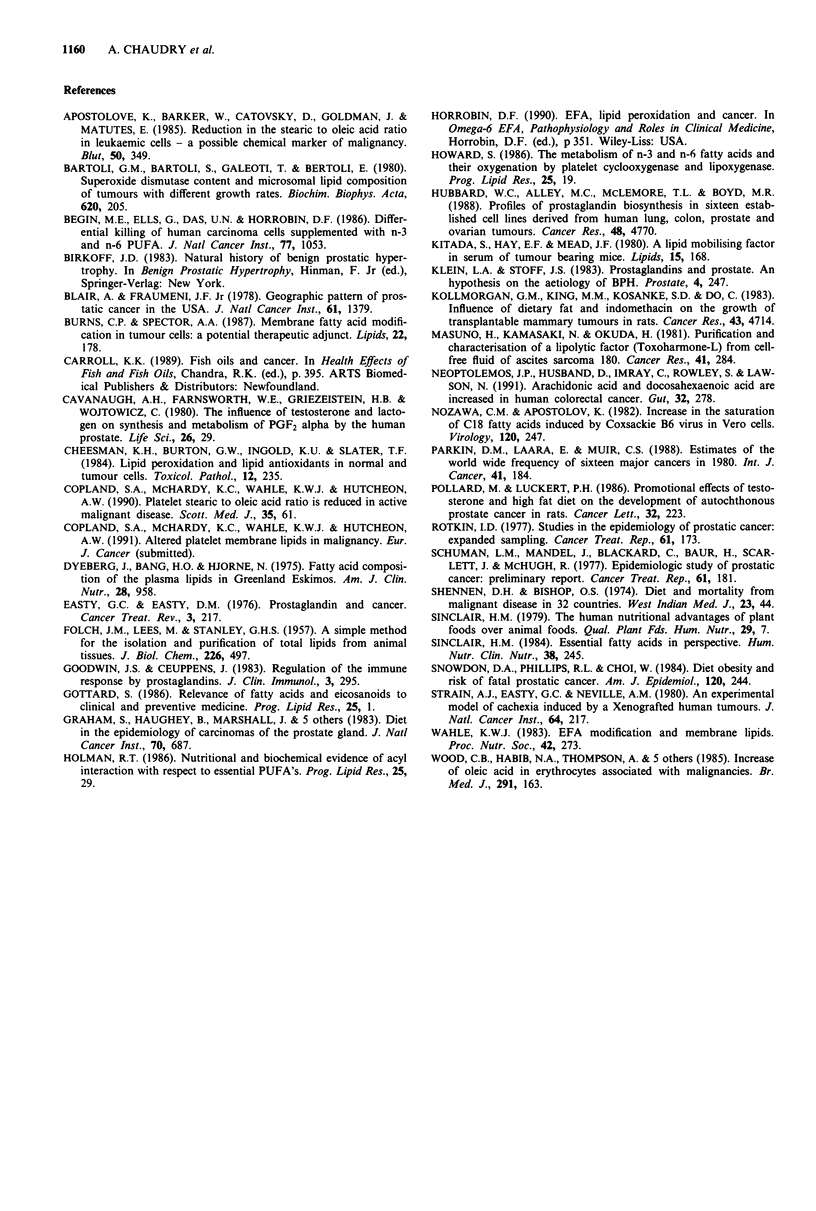# Essential fatty acid distribution in the plasma and tissue phospholipids of patients with benign and malignant prostatic disease.

**DOI:** 10.1038/bjc.1991.481

**Published:** 1991-12

**Authors:** A. Chaudry, S. McClinton, L. E. Moffat, K. W. Wahle

**Affiliations:** Department of Urology, Aberdeen Royal Infirmary, UK.

## Abstract

There is increasing evidence that essential fatty acids (EFA) may have a role to play in the aetiology of some types of cancer although their precise mode of action is unknown. Differences in the metabolism of EFA between patients with benign or malignant prostatic disease may help to elucidate their role in the latter. We have, therefore, measured the concentration of the essential fatty acids, and their metabolites, in the phospholipid fractions of both plasma and tissue, in patients with either benign or malignant prostatic disease. Comparison of the median concentration of fatty acids in each group (n = 10) revealed significant differences between them. The phospholipid component of total lipid was greater in malignant (P less than 0.04, unpaired t-test) than in benign tissue. The concentrations of linoleic acid (LA) and di-homo gamma linolenic acid (DGLA) in plasma and tissue were not different between the two groups of patients, but a significant reduction in arachidonic acid (ARA) (P less than 0.002, Mann-Whitney U-test) and docosapentaenoic acid (DPA) (P = 0.009) concentrations was observed in malignant tissue as compared to benign. Patients with malignant prostatic disease also had a significantly higher concentration of oleic acid in phospholipids from both plasma and prostatic tissue. The stearic to oleic acid ratio was similar in plasma but was significantly reduced in malignant tissue (P = 0.006). We suggest that the decreased arachidonic acid concentration in malignant tissue may be due to its increased metabolism, via the lipoxygenase and cyclooxygenase pathways to produce higher concentrations of eicosanoids, rather than an impairment in desaturase activity in situ.


Br. J. Cancer (1991), 64, 1157-1160 © Macmillan Press Ltd., 1991

### Essential fatty acid distribution in the plasma and tissue phospholipids of patients with benign and malignant prostatic disease

A. Chaudry[1], S. McClinton[1], L.E.F. Moffat[1] & K.W.J. Wahle[2]

[1]*Department of Urology, Aberdeen Royal Infirmary; and* [2]*Lipid Metabolism Unit, The Rowett Research Institute, Aberdeen, UK.*

**Summary** There is increasing evidence that essential fatty acids (EFA) may have a role to play in the aetiology of some types of cancer although their precise mode of action is unknown. Differences in the metabolism of EFA between patients with benign or malignant prostatic disease may help to elucidate their role in the latter. We have, therefore, measured the concentration of the essential fatty acids, and their metabolites, in the phospholipid fractions of both plasma and tissue, in patients with either benign or malignant prostatic disease.

Comparison of the median concentration of fatty acids in each group ($n = 10$) revealed significant differences between them. The phospholipid component of total lipids was greater in malignant ($P < 0.04$, unpaired *t*-test) than in benign tissue. The concentrations of linoleic acid (LA) and di-homo gamma linolenic acid (DGLA) in plasma and tissue were not different between the two groups of patients, but a significant reduction in arachidonic acid (ARA) ($P < 0.002$, Mann-Whitney U-test) and docosapentaenoic acid (DPA) ($P = 0.009$) concentrations was observed in malignant tissue as compared to benign.

Patients with malignant prostatic disease also had a significantly higher concentration of oleic acid in phospholipids from both plasma and prostatic tissue. The stearic to oleic acid ratio was similar in plasma but was significantly reduced in malignant tissue ($P = 0.006$).

We suggest that the decreased arachidonic acid concentration in malignant tissue may be due to its increased metabolism, via the lipoxygenase and cyclooxygenase pathways to produce higher concentrations of eicosanoids, rather than an impairment in desaturase activity *in situ*.

The prostate gland in the elderly male is often affected by benign hyperplasia or malignant neoplasia. Prostatic malignancy is the third most frequent male cancer in the industrialised countries (Parkin *et al.*, 1988). Benign prostatic hyperplasia affects the majority of the male population by the age of 80 years, with around 10% requiring surgical intervention (Birkoff, 1983). Despite the importance of these diseases, both in terms of mortality and morbidity, the aetiology remains unknown and there is no satisfactory treatment for hormone relapsed prostatic cancer. Recent epidemiological studies on carcinoma of the prostate gland report a positive relationship between the consumption of dietary fats and development of prostatic cancer in 32 countries (Shennen *et al.*, 1974). This association has been confirmed by almost all case controlled studies published to date (Rotkin *et al.*, 1977; Schuman *et al.*, 1977; Graham *et al.*, 1983; Snowdon *et al.*, 1984). These observations have lead to the suggestion that a high dietary fat intake may be a contributing factor in the initiation or development of this tumour (Blair *et al.*, 1978).

It is well established that the growth and development of the prostate gland requires the presence of sex hormones, particularly testosterone. Testosterone may act by altering the metabolism of fat in the body (Pollard *et al.*, 1986) and has been shown to regulate the synthesis, release and metabolism of prostaglandins (PGs) in the prostate gland (Klein *et al.*, 1983; Cavanaugh *et al.*, 1980). The synthesis of prostaglandins is initiated by the release of esterified arachidonic acid (ARA, C20:4n-6) from cellular phospholipids by phospholipase(s). ARA is the desaturated product of dietary linoleic acid (LA, C18:2n-6), an EFA which is the most prevalent polyunsaturated fatty acid component of membrane phospholipids (Gottard, 1986). The amount and composition of EFA in the membrane phospholipids can be modified by endocrine status and by both the amount and type of dietary fat ingested (Wahle, 1983; Howard, 1986); the presence of unusual fatty acids (i.e. *trans* or *cis* isomers of usual fatty acids) may affect EFA metabolism by inhibiting the desaturation pathways (Holman, 1986).

Cancer cells have been reported to have reduced concentrations of desaturated metabolites of LA such as gamma linolenic acid (GLA, C18:3n-6) and di-homo gamma linolenic acid (DGLA, C20:3n-6) (Bartoli *et al.*, 1980; Begin *et al.*, 1986) probably because of a reduced activity of delta-6 desaturase. This may cause an increase in LA concentrations with decreased conversion to ARA (Cheesman *et al.*, 1984; Burns *et al.*, 1987). However, a recent report (Neoptolemos *et al.*, 1991) has shown an increase in ARA and docosahexaenoic acid (DHA, C22:6n-3) concentrations in human colorectal cancer tissue. They suggest this increase is possibly due to a combination of enhanced desaturation of ARA precursor, reduced lipid peroxidation, and the increased production of prostanoids such as $PGE_2$. Clearly the question of whether cancer cells and malignant tissues have increased or decreased desaturase activities is controversial at present. The possibility that reported differences in desaturase activity reflect different types of tumours or differences between cell lines and tissues also requires clarification.

We report the fatty acid composition in plasma and prostatic tissue phospholipids of the patients with benign and malignant prostatic disease.

### Materials and methods

### *Patients and samples*

Patients admitted for prostatic surgery, for benign or malignant disease, were recruited to the study and written consent was obtained.

Blood samples were taken from fasting patients. Ten ml venous blood was dispensed in vacutainer tubes pretreated with EDTA (Becton & Dickinson, France), and centrifuged at 800 *g* for 30 min within 15 min of collection. Two ml aliquots of plasma were placed in separate tubes and stored immediately at −70°C.

Prostatic tissue was collected fresh at operation and was immediately frozen in liquid nitrogen and then stored at −70°C until analysed.

Correspondence: A. Chaudry, Department of Urology, Ward 44, Aberdeen Royal Infirmary, Foresterhill, Aberdeen AB9 2ZB, UK.
Received 11 February 1991; and in revised form 28 August 1991.

*Lipid analysis*

Total lipid extraction from all samples, including plasma and tissues, was carried out according to the method of Folch *et al.* (1975). All solvents used were AnalaR grade and had 0.005% BHT added as an antioxidant agent.

The phospholipids and neutral lipids of the total lipid extract were separated from each other by sequential elution with different ratios of chloroform and methanol through 'Sep-Pak' silica cartridges under vacuum (Waters Associates, USA). Only the phospholipids were analysed further as they are the most sensitive indicators of essential fatty acid changes. 2MeC16:0 as 5% of the weight of phospholipids was added as internal standard. Samples were methylated under reflux for 2 h in a heating block at 90°C with 2 ml methanolic hydrochloric acid. Fatty acid methyl esters (FAME) were extracted into di-ethyl ether, washed until neutral to litmus, taken to dryness under a stream of nitrogen, dissolved in 2 ml of hexane: di-ethyl ether (95:5 v/v) and eluted through heat activated silicic acid columns to remove contaminants and the resulting FAME analysed by capillary gas chromatography.

*Analysis of FAME*

FAME were analysed on a Pye Unicam, PU-4500 (Phillips, The Netherlands) gas chromatograph. Samples were injected onto a 25 m wall coated open tubular (WCOT) fused silica, CP Sil-5 SB, capillary column with 0.22 mm internal diameter (Chrompack International, The Netherlands). Helium, at a flow rate of 1 ml $min^{-1}$, was used as the carrier gas and pre-column split ratio was 92:1. Rise in column temperature was programmed at 180°C for 8 min, then 6°C $min^{-1}$ to 230°C and stable at 230°C for 7 min. Total run time for each sample was programmed to 25 min. Both injector and detector temperature was fixed at 300°C. FAME concentrations were determined using a flame ionisation detector, peaks were plotted and analysed on an SP4270 (Spectra-Physics, USA) plotter/integrator and were identified by their 'on column' retention time compared to those of known standards. Quantitation was done by comparing the area % of each FAME peak on the chromatogram with that of internal standard of known weight.

*Statistical analysis*

The total lipids and phospholipid content of plasma and tissue in the two groups were compared using the unpaired *t*-test. The median values and interquartile ranges for each fatty acid were then calculated and compared using the Mann Whitney U-test.

### Results

A total of 20 patients participated in the study. They were separated into either benign ($n = 10$) or malignant ($n = 10$) groups on the evidence obtained from histopathological examination of resected prostatic tissue.

**Figure 1** Total and phospholipids (means ± s.d.) recovered from plasma (mg $ml^{-1}$) and prostatic tissue (mg $g^{-1}$ wet weight) of patients with benign ($n = 10$) and malignant ($n = 10$) prostatic disease. *($P < 0.04$, compared with benign tissue).

*Plasma and tissue total lipid and phospholipid concentrations*

The mean values of total lipids and phospholipids from plasma and tissue in the benign and malignant groups are compared in Figure 1. The average total lipid content tended to be higher in malignant than in benign tissue samples, but this was not statistically significant. The mean values of plasma phospholipid concentrations in the benign and malignant groups were not significantly different. However, a significant difference ($P < 0.04$, unpaired *t*-test) was found in the phospholipid concentrations recovered from benign (mean ± s.d. = 5.37 ± 1.86 mg) and malignant (mean ± s.d. = 7.14 ± 1.77 mg) tissue samples.

*Plasma fatty acid concentrations (benign* vs *malignant)*

The fatty acid composition of plasma phospholipids in the benign and malignant group did not show any significant differences (Table I) except for an increased oleic acid (C18:1n-9) concentration in the malignant group ($P < 0.03$).

*Tissue fatty acid concentrations (benign* vs *malignant)*

The median concentrations of oleic acid were higher in malignant tissue when compared to benign tissue as is shown in Table II. While the concentrations of ARA and DPA were significantly higher ($P < 0.002$ and $P = 0.009$ respectively) in benign prostatic tissue when compared to the malignant tissue, no significant differences were observed in the concentrations of any other fatty acid.

**Table I** Fatty acid concentrations in plasma phospholipids of patients with prostatic disease (benign *vs* malignant) (figures expressed as percentage of total phospholipids)

| *Fatty acid* | *Benign (n = 10)* | *Malignant (n = 10)* | *Mann-Whitney P-value* |
|---|---|---|---|
| C16:0 | 25.53 (8.89)[a] | 26.67 (5.27) | N.S.[b] |
| C18:0 | 10.66 (1.48) | 10.76 (1.34) | N.S. |
| C18:1n-9 | 9.09 (2.75) | 11.09 (2.78) | 0.03 |
| C18:2n-6 | 19.67 (3.85) | 17.79 (4.51) | N.S. |
| C20:3n-6 | 2.56 (1.93) | 2.72 (1.35) | N.S. |
| C20:4n-6 | 8.78 (2.03) | 8.93 (1.84) | N.S. |
| C22:5 | 1.14 (0.42) | 1.20 (0.37) | N.S. |
| C22:6n-3 | 3.97 (1.28) | 3.82 (1.56) | N.S. |

[a]Median (interquartile range); [b]N.S. = not significant.

**Table II** Fatty acid concentrations in prostatic tissue phospholipids (benign *vs* malignant) (figures expressed as percentage of total phospholipids)

| *Fatty acid* | *Benign (n = 10)* | *Malignant (n = 10)* | *Mann-Whitney P-value* |
|---|---|---|---|
| C16:0 | 20.77 (4.32)[a] | 21.55 (2.03) | N.S.[b] |
| C18:0 | 12.46 (1.84) | 11.90 (1.54) | N.S. |
| C18:1n-9 | 14.56 (3.73) | 21.32 (7.72) | 0.002 |
| C18:2n-6 | 7.41 (2.47) | 6.16 (2.44) | N.S. |
| C20:3n-6 | 1.87 (0.77) | 1.72 (0.36) | N.S. |
| C20:4n-6 | 15.55 (2.54) | 11.33 (4.12) | 0.002 |
| C22:5 | 2.83 (0.71) | 2.06 (0.76) | 0.009 |
| C22:6n-3 | 3.11 (0.71) | 3.22 (0.87) | N.S. |

[a]Median (interquartile range); [b]N.S. = not significant.

**Table III** Stearic acid to oleic acid ratio[a]

| *Sample* | *Benign (n = 10)* | *Malignant (n = 10)* | *P-value* |
|---|---|---|---|
| Plasma | 1.25 (0.37) | 0.99 (0.23) | N.S.[b] |
| Tissue | 0.90 (0.23) | 0.60 (0.20) | 0.006 |

[a]Median (interquartile range); [b]N.S. = not significant

*Stearic acid to oleic acid ratio*

The median values of the ratios between stearic acid (C18:0) and oleic acid for all the patients, in both groups, are shown in Table III. No differences were seen in the ratio of these fatty acids in the plasma fatty acid pool of benign or malignant patients. However, this ratio is significantly lower in malignant than in benign prostatic tissue.

### Discussion

To our knowledge, this is the first study which clearly indicates a variation of fatty acid composition in patients with benign and malignant prostatic disease.

The concentration of LA, the main essential fatty acid, was not different, in either plasma or prostatic tissue, between the two groups. This is despite the expected increased utilisation of La to build the cell membranes of rapidly dividing cancer cells. However, many reports have indicated that growing tumours are able to mobilise fatty acids from host stores to meet the increased demand for membrane growth (Kitada *et al.*, 1980; Strain *et al.*, 1980). It has also been reported that a fat mobilising factor may be secreted by some tumour cells in order to ensure an adequate supply of fatty acids (Masuno *et al.*, 1981).

LA is further desaturated to form GLA by delta-6 desaturase. Further elongation of this fatty acid produced DGLA, the precursor of the I-series prostaglandins; delta-5 desaturation then results in the formation of ARA, the precusor of the II-series prostaglandins (Wahle, 1983). Delta-6-desaturase activity is reportedly reduced in cancer cells because of the low concentrations of GLA and DGLA found in these cells which were regarded as DGLA deficient (Horrobin, 1990). In contrast to these observations, our results show no difference in the tissue concentrations of LA and DGLA between the two groups of patients. This does not suggest a reduction in delta-6 desaturase activity in prostatic cancer tissue. The possibility that cancer cell lines and cancer tissue may differ with regard to their desaturase activities is worthy of investigation. Determinations of desaturase activity *per se*, in microsomal preparations from benign and malignant tissues, rather than by the fatty acid end-product concentration in the tissue are currently being elucidated. Our suggestion that the delta-6 desaturase activities in malignant and benign prostatic tissue do not differ significantly is also at variance with the findings of Neoptolemos *et al.* (1991) who reported an increase in ARA and DHA concentrations in malignant colonic tissue and suggested this could be due, in part, to an enhancement desaturase (delta-6, delta-5) activity in human colonic cancer cells.

In the present studies, despite similar DGLA concentrations in malignant and benign prostatic tissue, ARA concentrations were significantly reduced in malignant tissue and DHA concentrations were again similar in both tissues. The lack of agreement between our observations and those of Neoptolemos *et al.* (1991) may be a reflection of the different types of tumour studied.

One possible mechanism to explain the significant decrease in ARA and DPA concentrations in malignant compared with benign tissue in the present studies would be an impairment of delta-5 desaturase enzyme activity in malignant tissues. Such an impairment has been postulated to occur in healthy Greenland Eskimos (Dyerberg *et al.*, 1975; Sinclair, 1979) on the basis of their increased plasma DGLA and DHA concentrations when compared with those in Europeans. However, the concentrations of these fatty acids did not differ between tissues in the present studies. DGLA could be metabolised through the cyclooxygenase pathway to form $PGE_1$ but many cancers, including prostatic cancer, produce excessive amounts of $PGE_2$ from ARA yet are unable to make $PGE_1$ (Easty *et al.*, 1976). Increased metabolism of ARA to cyclooxygenase, and possibly lipoxygenase, products could explain the lower ARA concentration in malignant tissue in the present study. Preliminary studies *in vitro* have shown that malignant prostatic tissue has a greatly increased capacity for eicosanoid synthesis from radiolabelled ARA compared with the benign tissue (Chaudry & Wahle, 1991, unpublished data). This lends support to the above explanation and suggests a possible central role for ARA in the aetiology of cancer.

Metabolism of ARA can proceed by pathways other than eicosanoid synthesis, for example by elongation to adrenic acid (C22:4n-6) followed by desaturation to DPA. This seems unlikely to be the explanation for the reduced ARA in malignant tissue given the observed reduction of DPA in this tissue.

Increased ARA metabolism via the cyclooxygenase pathway seems at present the most likely explanation for the reduced concentration of ARA in malignant tissue which we have demonstrated. $PGE_2$, the main cyclooxygenase product of ARA is known to be a tumour promoter (Goodwin *et al.*, 1983) and is often found in high concentrations in cancer cells (Hubbard *et al.*, 1988). This may have therapeutic implications as the cancer promoting effects of $PGE_2$ can be inhibited by cyclooxygenase inhibitors such as Indomethacin (Kollmorgen *et al.*, 1983). Indeed feeding fish oils (n-3 fatty acids) to rats with prostatic tumours has been reported to be beneficial, as the desaturated metabolites of the n-3 family competitively inhibit the production of ARA derived eicosanoids (see Carroll, K., 1989 for recent review).

We found the ratio between stearic acid and oleic acid in malignant prostatic tissue to be significantly decreased. A reduction of stearic to oleic acid ratio in the platelets of patients with active malignancies (Copland *et al.*, 1990) has previously been reported. Similar findings were observed in the peripheral blood cells of patients with chronic leukaemia (Apostolov *et al.*, 1985) and in the circulating erythrocytes of patients with solid tumours (Wood *et al.*, 1985). The reason for the decreased stearic to oleic acid ratio in malignant tissue is not clear at present. It has been reported that the affinity of fatty acids for delta-6 desaturation is dependent on the number of double bonds. Linoleic acid, for example, has more affinity for delta-6 desaturase than oleic acid (Sinclair, 1984). Thus the desaturation of LA abolishes further metabolism of oleic acid and oleic acid is desaturated to eicosatrienoic acid (C20:3n-9) only in EFA deficiency (Sinclair, 1984). Reduced metabolism of oleic acid will obviously result in the reduction of stearic to oleic acid ratio.

It has been suggested that this reduced ratio in erythrocytes is a useful diagnostic marker for malignancies (Wood *et al.*, 1985). However, a similar decrease in stearic:oleic acid ratio has been observed in cells infected with Coxsackie and Herpes simplex type 2 viruses (Nozawa *et al.*, 1982) and in patients with non-malignant diseases (Copland *et al.*, 1991). We conclude from these observations that the stearic:oleic acid ratio probably reflects abnormalities in fatty acid metabolism in general, and may not be a useful index in the diagnosis and postoperative monitoring of patients with cancer.

In conclusion, our results show that the concentration of arachidonic acid in malignant prostatic tissue is significantly lower than that found in benign tissue and we suggest that this may be due to increased ARA metabolism, possibly to form prostaglandins (as supported by our preliminary findings), high concentrations of which are often found in malignant tissue. Further investigation of the mechanisms which modify EFA metabolism in malignant as compared with benign prostatic tissue may clarify the role of the EFA/prostanoids in the aetiology of prostatic disease.

### References

APOSTOLOVE, K., BARKER, W., CATOVSKY, D., GOLDMAN, J. & MATUTES, E. (1985). Reduction in the stearic to oleic acid ratio in leukaemic cells – a possible chemical marker of malignancy. *Blut*, **50**, 349.

BARTOLI, G.M., BARTOLI, S., GALEOTI, T. & BERTOLI, E. (1980). Superoxide dismutase content and microsomal lipid composition of tumours with different growth rates. *Biochim. Biophys. Acta*, **620**, 205.

BEGIN, M.E., ELLS, G., DAS, U.N. & HORROBIN, D.F. (1986). Differential killing of human carcinoma cells supplemented with n-3 and n-6 PUFA. *J. Natl Cancer Inst.*, **77**, 1053.

BIRKOFF, J.D. (1983). Natural history of benign prostatic hypertrophy. In *Benign Prostatic Hypertrophy*, Hinman, F. Jr (ed.), Springer-Verlag: New York.

BLAIR, A. & FRAUMENI, J.F. Jr (1978). Geographic pattern of prostatic cancer in the USA. *J. Natl Cancer Inst.*, **61**, 1379.

BURNS, C.P. & SPECTOR, A.A. (1987). Membrane fatty acid modification in tumour cells: a potential therapeutic adjunct. *Lipids*, **22**, 178.

CARROLL, K.K. (1989). Fish oils and cancer. In *Health Effects of Fish and Fish Oils*, Chandra, R.K. (ed.), p. 395. ARTS Biomedical Publishers & Distributors: Newfoundland.

CAVANAUGH, A.H., FARNSWORTH, W.E., GRIEZEISTEIN, H.B. & WOJTOWICZ, C. (1980). The influence of testosterone and lactogen on synthesis and metabolism of $PGF_2$ alpha by the human prostate. *Life Sci.*, **26**, 29.

CHEESMAN, K.H., BURTON, G.W., INGOLD, K.U. & SLATER, T.F. (1984). Lipid peroxidation and lipid antioxidants in normal and tumour cells. *Toxicol. Pathol.*, **12**, 235.

COPLAND, S.A., MCHARDY, K.C., WAHLE, K.W.J. & HUTCHEON, A.W. (1990). Platelet stearic to oleic acid ratio is reduced in active malignant disease. *Scott. Med. J.*, **35**, 61.

COPLAND, S.A., MCHARDY, K.C., WAHLE, K.W.J. & HUTCHEON, A.W. (1991). Altered platelet membrane lipids in malignancy. *Eur. J. Cancer* (submitted).

DYEBERG, J., BANG, H.O. & HJORNE, N. (1975). Fatty acid composition of the plasma lipids in Greenland Eskimos. *Am. J. Clin. Nutr.*, **28**, 958.

EASTY, G.C. & EASTY, D.M. (1976). Prostaglandin and cancer. *Cancer Treat. Rev.*, **3**, 217.

FOLCH, J.M., LEES, M. & STANLEY, G.H.S. (1957). A simple method for the isolation and purification of total lipids from animal tissues. *J. Biol. Chem.*, **226**, 497.

GOODWIN, J.S. & CEUPPENS, J. (1983). Regulation of the immune response by prostaglandins. *J. Clin. Immunol.*, **3**, 295.

GOTTARD, S. (1986). Relevance of fatty acids and eicosanoids to clinical and preventive medicine. *Prog. Lipid Res.*, **25**, 1.

GRAHAM, S., HAUGHEY, B., MARSHALL, J. & 5 others (1983). Diet in the epidemiology of carcinomas of the prostate gland. *J. Natl Cancer Inst.*, **70**, 687.

HOLMAN, R.T. (1986). Nutritional and biochemical evidence of acyl interaction with respect to essential PUFA's. *Prog. Lipid Res.*, **25**, 29.

HORROBIN, D.F. (1990). EFA, lipid peroxidation and cancer. In *Omega-6 EFA, Pathophysiology and Roles in Clinical Medicine*, Horrobin, D.F. (ed.), p 351. Wiley-Liss: USA.

HOWARD, S. (1986). The metabolism of n-3 and n-6 fatty acids and their oxygenation by platelet cyclooxygenase and lipoxygenase. *Prog. Lipid Res.*, **25**, 19.

HUBBARD, W.C., ALLEY, M.C., MCLEMORE, T.L. & BOYD, M.R. (1988). Profiles of prostaglandin biosynthesis in sixteen established cell lines derived from human lung, colon, prostate and ovarian tumours. *Cancer Res.*, **48**, 4770.

KITADA, S., HAY, E.F. & MEAD, J.F. (1980). A lipid mobilising factor in serum of tumour bearing mice. *Lipids*, **15**, 168.

KLEIN, L.A. & STOFF, J.S. (1983). Prostaglandins and prostate. An hypothesis on the aetiology of BPH. *Prostate*, **4**, 247.

KOLLMORGAN, G.M., KING, M.M., KOSANKE, S.D. & DO, C. (1983). Influence of dietary fat and indomethacin on the growth of transplantable mammary tumours in rats. *Cancer Res.*, **43**, 4714.

MASUNO, H., KAMASAKI, N. & OKUDA, H. (1981). Purification and characterisation of a lipolytic factor (Toxoharmone-L) from cell-free fluid of ascites sarcoma 180. *Cancer Res.*, **41**, 284.

NEOPTOLEMOS, J.P., HUSBAND, D., IMRAY, C., ROWLEY, S. & LAWSON, N. (1991). Arachidonic acid and docosahexaenoic acid are increased in human colorectal cancer. *Gut*, **32**, 278.

NOZAWA, C.M. & APOSTOLOV, K. (1982). Increase in the saturation of C18 fatty acids induced by Coxsackie B6 virus in Vero cells. *Virology*, **120**, 247.

PARKIN, D.M., LAARA, E. & MUIR, C.S. (1988). Estimates of the world wide frequency of sixteen major cancers in 1980. *Int. J. Cancer*, **41**, 184.

POLLARD, M. & LUCKERT, P.H. (1986). Promotional effects of testosterone and high fat diet on the development of autochthonous prostate cancer in rats. *Cancer Lett.*, **32**, 223.

ROTKIN, I.D. (1977). Studies in the epidemiology of prostatic cancer: expanded sampling. *Cancer Treat. Rep.*, **61**, 173.

SCHUMAN, L.M., MANDEL, J., BLACKARD, C., BAUR, H., SCARLETT, J. & MCHUGH, R. (1977). Epidemiologic study of prostatic cancer: preliminary report. *Cancer Treat. Rep.*, **61**, 181.

SHENNEN, D.H. & BISHOP, O.S. (1974). Diet and mortality from malignant disease in 32 countries. *West Indian Med. J.*, **23**, 44.

SINCLAIR, H.M. (1979). The human nutritional advantages of plant foods over animal foods. *Qual. Plant Fds. Hum. Nutr.*, **29**, 7.

SINCLAIR, H.M. (1984). Essential fatty acids in perspective. *Hum. Nutr. Clin. Nutr.*, **38**, 245.

SNOWDON, D.A., PHILLIPS, R.L. & CHOI, W. (1984). Diet obesity and risk of fatal prostatic cancer. *Am. J. Epidemiol.*, **120**, 244.

STRAIN, A.J., EASTY, G.C. & NEVILLE, A.M. (1980). An experimental model of cachexia induced by a Xenografted human tumours. *J. Natl. Cancer Inst.*, **64**, 217.

WAHLE, K.W.J. (1983). EFA modification and membrane lipids. *Proc. Nutr. Soc.*, **42**, 273.

WOOD, C.B., HABIB, N.A., THOMPSON, A. & 5 others (1985). Increase of oleic acid in erythrocytes associated with malignancies. *Br. Med. J.*, **291**, 163.